# In vivo toxicity and antitumor activity of essential oils extract from agarwood (*Aquilaria crassna*)

**DOI:** 10.1186/s12906-016-1210-1

**Published:** 2016-07-22

**Authors:** Saad Sabbar Dahham, Loiy E. Ahmed Hassan, Mohamed B. Khadeer Ahamed, Aman Shah Abdul Majid, Amin Malik Shah Abdul Majid, Nik Noriman Zulkepli

**Affiliations:** EMAN Research and Testing Laboratory, School of Pharmaceutical Sciences, Universiti Sains Malaysia, Penang, 11800 Malaysia; Department of Pharmacology, School of Medical Sciences, Quest International University, Perak, Malaysia; Center of Excellence Geopolymer and Green Technology (CEGeoGTech), Faculty of Engineering Technology (FETech), Universiti Malaysia Perlis (UniMAP), UniCITI Alam Campus, 02100 Padang Besar, Perlis Malaysia

**Keywords:** Agarwood, Essential oils, Oral toxicity, Anti-tumor, Nude mice

## Abstract

**Background:**

*Aquilaria crassna* has been used in traditional Asian medicine to treat vomiting, rheumatism, asthma, and cough. Furthermore, earlier studies from our laboratory have revealed that the essential oil extract from agarwood inhibited colorectal carcinoma cells. Despite of the wide range of ethno-pharmacological uses of agarwood, its toxicity has not been previously evaluated through systematic toxicological studies. Therefore, the potential safety of essential oil extract and its *in vivo* anti-tumor activity had been investigated.

**Methods:**

In the acute toxicity study, Swiss female mice were given a single dose of the essential oil extract at 2000 mg/kg/day orally and screened for two weeks after administration. Meanwhile, in the sub-chronic study, two different doses of the extract were administered for 28 days. Mortality, clinical signs, body weight changes, hematological and biochemical parameters, gross findings, organ weights, and histological parameters were monitored during the study. Other than that, in vivo anti-tumor study was assessed by using subcutaneous tumors model established in nude mice.

**Results:**

The acute toxicity study showed that the LD_50_ of the extract was greater than 2000 mg/kg. In the repeated dose for 28-day oral toxicity study, the administration of 100 mg/kg and 500 mg/kg of essential oil per body weight revealed insignificant difference in food and water intakes, bodyweight change, hematological and biochemical parameters, relative organ weights, gross findings or histopathology compared to the control group. Nevertheless, the essential oil extract, when supplemented to nude mice, caused significant growth inhibition of the subcutaneous tumor of HCT 116 colorectal carcinoma cells.

**Conclusion:**

Collectively, the data obtained indicated that essential oil extract from agarwood might be a safe material, and this essential oil is suggested as a potential anti-colon cancer candidate.

## Background

There is some truth to the old adage that the therapeutic use of natural products and their derivatives is as old as the human race. The ancient civilizations provided written evidences for the use of natural sources to treat various illnesses. In fact, the oldest description of medical document dated 4000 years ago, depicted on a Sumerian clay table with a list of the most valuable medicinal plants [1]. The knowledge and historical experiences of medicinal plant have been passed from one generation to the next, interpreted as accumulative body and form the basis of traditional and modern medicine.

According to the World Health Organization (WHO), about 80 % of population in the developing countries relies on traditional herbal medicine for primary health care needs. Recently, a wide range of plant-derived phytochemical components and their synthetic derivatives have been suggested for cancer treatment. Moreover, it has been estimated that 25 %-48 % of the currently approved therapies by the Food and Drug Administration (FDA) are derived from plants. Surprisingly, more than 10,000 phytochemicals have been identified and used in cancer treatment due to their privileged structure and broad spectrum of biological activities [2, 3]. Phytochemicals, such as paclitaxel, vincristine, and camptothecin, have become major and significant sources for chemotherapy in cancer treatment protocols, which provided the most successful alternative and complementary anti-cancer regimen [4]. Also, there is the worldwide green revolution that is generated from the notion that medicinal plants are safer and less harmful to the human body than synthetic drugs, and besides, there is wide consensus that the advantage of natural components over synthetic compounds is less toxic based on their long term use [5].

Nevertheless, failing to identify the chemical profile of plants and the safety assessment may lead to deleterious effects on health. Furthermore, it has been well-documented that from about 1,500,000 plants investigated, most of them contained toxic constituents, indicating that medicinal plants should be used with precaution and toxicology investigation is indeed vital to increase the knowledge of ethnopharmacological uses of plants [6].

The genus of Aquilaria (Thymelaeceae) is comprised of approximately 15 species distributed across the rain forests of Southeast Asia, where only 8 species were reported for agarwood formation. Agarwood is also known as gaharu in Malaysia, eaglewood and krissana in Thailand, Oudh in Middle East, chen-xiang in Chinese, and jin-koh in Japan [7]. In general, species like *Aqularia malaccensis* (agallocha), *A. crassna*, and *A. sinensis* have been widely explored for their chemical constituents (resin and essential oils), while less focus had been given to their pharmacological activities. The products of Aqularia, including Agarwood oil, leaves, and seeds, were prescribed in traditional Asian medicine to treat vomiting, rheumatism, and asthma. In Malaysian traditional medicine, (gaharu) used to be mixed with coconut oil as an ointment to treat body pain [8]; (jin-koh) has been used for traditional medicine in Japan as a sedative or tranquilizer in detoxifying the body and maintaining stomach health [9]. In addition, much attention has been paid to the phytochemical investigation and the characterization of the active components from this plant in these recent years. *Aquilaria crassna*, in addition, has been identified as convenient and useful sources of bioactive constituents, such as phenolic, flavonoids, benzophenones, xanthones, and Sesquiterpenes [10]. In fact, several studies have looked into the biological activities of *Aquilaria crassna*. In particular, it seemed to possess anti-ischemic [11], anti-fungal [12], and anti-bacterial effects [13]. Earlier communications from this laboratory reported the extraction and the purification of the essential oil derived from *Aquilaria crassna* and the active principle was identified as well. The biological investigation revealed potent antioxidant and *in vitro* anti-cancer properties against colorectal carcinoma cells (HCT 116) and pancreatic cancer cells (MIA PaCa-2), which had been mediated via apoptotic mechanism [14–16].

Despite of the broad spectrum of biological activities, no study had reported on the toxicological profiles of *Aquilaria crassna* essential oil. With this in mind, the present study had been designed to bridge the informational gaps with the following objectives:

- To study the acute and the sub-chronic oral toxicity profile of *A. crassna* essential oil in Swiss mice.
- To study the anti-tumor efficacy of *A.crassna* essential oil by employing subcutaneous tumor model of human colorectal cancer.

## Methods

### Agarwood material and extraction of essential oil

Fresh sample of *A. crassna* stem bark was obtained from a local farm in Kajang, Selangor, Malaysia, in the year 2014. In addition, flowers and leaves with twigs were collected for taxonomical authentication and were deposited at the School of Biological Sciences, USM (Ref. No. USM/122083). The bark of *A. crassna* was cleaned thoroughly, cut into small slices, and ground mechanically. The ground material of the stem bark (500 g) was macerated with distilled water (5 L) at room temperature (25 ± 2 °C) for 1 week. Finally, hydrodistillation was employed on the extract for 48 h at boiling temperature of water. Essential oil (EOs) was obtained by using the Clevenger-type apparatus. The EO was collected as pale-yellow liquid with 12.6 g yield.

### Toxicity studies

#### Experimental animals

Male and female Swiss mice at 8-12weeks (25-30 g) were obtained from the animal house facility of USM. The animals were kept in the transit animal house at the School of Pharmaceutical Sciences to acclimatize them for five days. During this period, the mice were kept in ventilated cages at 12 h light cycle with continuous supply of food and water. The procedures described in this study were approved by the USM Animal Ethics Committee with the reference number of USM/2014/ (94) (674).

#### Acute toxicity in mice

A limit test was performed according to the Fixed Dose Procedure of the OECD guideline 425 to evaluate the oral toxicological profiling of the essential oil extract [17]. Five healthy Swiss female mice were randomly selected, marked to permit individual identification, and kept in their cages for at least 5-7 days prior to dosing for acclimatization to the laboratory conditions. 2000 mg/kg body weight of extract was administrated to overnight fasted mice in a single dose via oral gavage (the maximum administered volume was 10 ml/kg). After the essential oil was administered, food, but not water, was withheld for 3-4 hrs. According to the guideline, one animal at a time was treated with 2000 mg/kg and the animal was monitored for 48 hours. When the animal did not die, then another four animals were treated at the same dose and were monitored for 14 days. In case of death of the first animal within 48 hours of treatment, the main test protocol was adhered. All animals were weighed on 0, 7th, and 14th days and were observed for any signs or symptoms of toxicity and/or mortality. The visual observations were recorded, including the changes in skin and fur, eyes and mucous membranes, as well as in the behavioral pattern, such as alertness and positioning of the animals.

#### Sub-chronic toxicity in mice

A sub-chronic repeated dose (28 days) study in mice was conducted according to the OECD 407 guideline [18]. Healthy male and female Swiss mice were randomly divided into three groups, comprising of 10 animals in each group. The essential oil was emulsified in Tween-80 at 1 % before it was administrated to the animals. The essential oil extract was administered orally and daily for 28 days in single doses of 500 mg/kg (group I) and 1000 mg/kg (group II), whereas the control (group III) received only aqueous with (1 % Tween-80). Body weight was recorded at 0, 7th, 14th, and 28th days. Along with food and water consumptions, toxic manifestations and mortality were also monitored daily throughout the study period. At the end of the experiment, all mice were anesthetized via carbon dioxide inhalation and their blood samples were collected via cardiac puncture into non-heparinized and EDTA containing tubes for biochemical and hematological analyses, respectively. After blood collection, the animals were sacrificed via cervical dislocation for isolation of organs to observe histopathological changes. The liver, kidney, lung, brain, spleen, and heart were excised, weighed using analytical lab balance (Mettler-Toledo AX-204, Japan), examined macroscopically, and finally, fixed in 10 % buffered neutral formalin for histopathological examination.

#### Hematology indexes

The blood was analyzed within one hour by using Sysmex-XT-1800 Automated Hematology Analyzer, Germany Analyses, performed at Gribbles lab (Pinang Malaysia). The hematology indexes included red blood cell (RBC), white blood cell (WBC), neutrophil, lymphocyte, eosinophil, monocyte, basophil, hemoglobin concentration (Hb), hematocrit (Ht), mean corpuscular volume (MCV), mean corpuscular hemoglobin (MCH), mean corpuscular hemoglobin concentration (MCHC), and platelet count (Plt).

#### Clinical biochemistry indexes

The blood samples were centrifuged immediately after collection at 3000 rpm for 15 min, and the supernatant was collected as the serum. The clinical biochemistry indexes were measured within 1 h by using an automated Olympus 640 Biochemistry Analyzer, Japan Analysis, performed at Gribbles lab. (Pinang, Malaysia). With that, alkaline phosphatase (ALP), aspartate aminotransferase (AST), alanine aminotransferase (ALT), lactate dehydrogenase, creatin phosphokinase, total protein, total albumin, albumin/globulin ratio, phosphorus, calcium, sodium, potassium, chloride, as well as total and conjugated bilirubin, were measured.

#### Histopathological study

As for histopathological study, 3 randomly selected mice from each experimental group were euthanized and the named organs were harvested and fixed in 10 % buffered neutral formalin for 48 hours, and then with bovine solution for 6 hours, and processed for paraffin embedding. Sections of 5 μm thickness were taken by using a microtome, processed in alcohol-xylene series, and were stained with both hematoxylin and eosin.

### In vivo anti-tumor model

#### Experimental animals

Athymic NCR nu/nu nude mice were obtained from Taconic Farms Inc., USA. According to the supplier, the autosomal recessive nude gene in homozygous (nu/nu) mice caused the lack of fur and an abnormal thymus. The deficiency in T cell function allowed athymic mice to accept and develop xenografts tumor model. The mice of the same gender were housed in specific pathogen free (SPF) cages supplied with high efficiency particulate air (HEPA) filters. Free access to autoclaved food and water was provided and the autoclaved bedding was changed twice weekly. The procedures were approved by the USM Animal Ethics Committee with the reference number USM/2014/ (94) (672).

#### In vivo anti-tumor activity

Twenty four nude mice aged 6–8 weeks with an average weight of 25 g were injected subcutaneously in the right flank with 5 × 10^6^ HCT 116 cells in 150 μl RPMI. After 7–10 days of tumor initiation, the animals were divided randomly into 4 groups of 6–8 animals. Group 1 received 0.2 ml of distilled water with 0.1 % tween-80 as control, while Groups 2, 3, and 4 received oral treatments with 200, 100, and 50 mg/kg bodyweight of essential oil extract. The tumor size and the body weight were recorded before starting the treatment and at 5 days interval until the end of the experiment. Treatment of animals was performed orally via oral gavages (wt/wt) once a day for a period of 6-8 weeks. The tumor size was calculated as described previously [19, 20] by applying the formula: Tumor volume (mm^3^) = (((W + L) / 2) ^ 3) × 2; where W is the width and L is the length. For tumors with more than one lobe, the tumor size was calculated through the summation of the size of the individual lobes. Also, the following value was calculated: % ∆T/∆C, where, ∆T = T − ∆0 and ∆C = C − ∆0 (∆0 is the average tumor volume at the beginning of the treatment, whereas T and C are the tumor volumes at a specified day for treated and control groups, respectively). Normally, the ∆T/∆C value in percent is used as an indication of anti-tumor effectiveness, and a value of ∆T/∆C ≤ 42 % is considered as significant anti-tumor activity by the Division of Cancer Treatment, NCI, NIH [21]. At the end of the experiment, the animals were euthanized with CO2, and followed by cervical dislocation. The subcutaneous tumors were excised and the tumors were preserved in 4 % paraformaldehyde. Cross sections were prepared and stained with eosin/hematoxylin. The extent of apoptosis/necrosis and the number of blood vessels were examined in 25 microscopic fields at 20x magnification.

#### Statistical analysis

The results are presented as mean ± SD. The differences between groups were compared via One-way ANOVA, and were considered significant at P < 0.05. The statistical analysis was carried out by using GraphPad prism version 6.

## Results

### Acute toxicity study

The acute toxicity test allowed the estimation of median lethal dose (LD_50_), which represented the dose that killed 50 % of the tested population, which had been used to appreciate the toxicity of the samples. The study also provided useful information concerning the effect of acute exposure of test animals to high doses of extracts under investigation. Treatment of female and male Swiss mice with essential oil of *A.crassna* did not produce treatment-related mortality at the limit test dose (2000 mg/kg), and besides, throughout the 14 days observation period, no significant changes had been discovered in the behavior, such as apathy, hyperactivity, dizziness, vomiting, diarrhea, excessive salivation, loss of fur, anxiety, convulsions, lethargy, and morbidity, among the tested animals. Furthermore, no abnormal changes attributable to treatment had been noticed in body weights and treatment related changes like respiration rate and heart rate. Thus, EOs had been found to be safe at the dose level of 2000 mg/kg and therefore, the LD_50_ value for oral toxicity had been considered to be more than 2000 mg/kg.

### Sub-chronic toxicity study

**Fig. 1:**
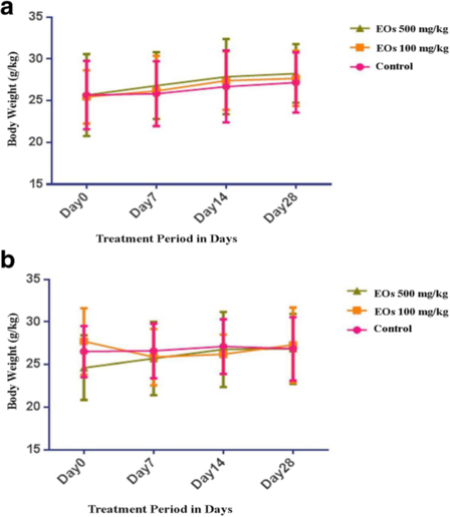
Body weight changes of male (**a**) and female (**b**) Swiss mice during the 28-day toxicological assessment. The vehicle, water (10 ml/kg/day), was administered to mice in the control group. No significant differences were detected between the treated (100 and 500 mg/kg. All values are expressed as the mean ± SD (n = 10)

The effect of 28-day oral administration of the EOs on general behavior, hematological, and biochemical parameters in Swiss mice showed no signs of toxicity, such as piloerection, diarrhea, sedation, loss of fur, and alterations in locomotors activity or mortality, as recorded during the 28 consecutive days of treatment via oral route with the EOs at the doses of 100 and 500 mg/kg. The body weight gain of both genders was not affected by EOs treatment compared to control group (Fig. 1). However, slight changes in food and water consumptions in both sexes were observed in treated groups during the treatment period compared to control group.

### Hematological and biochemical parameters

The results of the hematological profile of mice exposed to sub-acute treatment with the EOs of *A.crassna* (100 and 500 mg/ml) and control group are shown in Table 1. However, no changes had been recorded in the hematological indices in either sex. Similarly, the biochemical parameters are represented in Table 2, where no significant deviations had been found between the treated groups and the control of parameters tested.

**Table 1:** Effects of the sub chronic oral administration of EOs on hematological parameters in Swiss mice

Parameters	Control		EOs 100 mg/kg		EOs 500 mg/kg	
	Male	Female	Male	Male	Male	Female
Hemoglobin (g/dL)	17.00 ±1.25	15.00 ± 1.50	16.00 ± 1.55	16.50±2.52	15.40±2.70	16.00 ±2.62
Total Red Blood Cell (10^12^/L)	11.70±0.24	10.19 ± 0.25	11.80±0.25	11.62±0.14	11.67±0.31	13.50±0.50*
Red cell distribution width (%)	22.10±1.33	20.30±0.90	22.30±1.11	20.39±0.11	23.00±1.25	21.94±1.84
Total White Blood Cell (10^9^/L)	9.30±2.51	8.98 ± 1.22	9.50±1.69	8.61±2.70	8.44±1.95	6.93±1.00*
Neutrophils (%)	3.90±2.11	2.70 ± 2.29	3.50±2.99	2.50±2.11	2.60±2.11	3.90±2.81
Lymphocytes (%)	6.40±2.10	5.88 ± 2.00	6.50±2.13	6.33±2.14	10.70±2.52	6.52±2.12
Eosinophils (%)	0.05±0.02	0.00 ± 0.00	0.03±0.09	0.08±0.05	0.03±0.25	0.08±0.07
Monocytes (%)	0.60±0.10	0.60 ± 0.40	0.33±0.50	0.80±0.45	0.70±0.40	0.70±0.50
Basophils (%)	0.00 ± 0.00	0.01 ± 0.02	0.00 ± 0.00	0.00 ± 0.00	0.01 ± 0.05	0.50 ± 0.05
Packed Cell Volume (%)	0.52±0.5	0.75 ± 0.58	0.55±0.42	0.70±0.50	0.47±0.44	0.72±0.25
Mean Corpuscular (fl)	44.00±0.63	50.00 ± 0.76	47.00±0.40	51.00±0.26	44.00±0.60	53.40±0.12
Mean Corpuscular Hb (pg)	15.00±0.52	18.40 ± 0.18	15.00±0.50	17.99±0.55	14.00±0.82	15.49±0.49
Platelet count(10^9^/L)	1419.10±96.69	1293.00±85.49	1221.50±89..27	1167.70±30.26	1292.00±10..33	1375.80±28.20

Values are presented as mean ± SD, (*P < 0.05)

**Table 2:** Effects of the sub chronic oral administration of EOs on hematological parameters in Swiss mice

Parameters	Control		EOs 100 mg/kg		EOs 500 mg/kg	
	Male	Female	Male	Female	Male	Female
Total protein (g/dL)	6.00 ± 0.89	5.30 ± 1.10	5.67 ± 1.61	5.59± 1.88	6.6 0± 1.38	6.00 ± 1.99
Albumin (g/dL)	3.05 ± 0.68	3.33 ± 0.58	3.00 ± 0.55	4.00 ± 1.18	4.55 ± 058	3.89 ± 0.50
Globulin (g/dL)	3.32 ± 0.22	2.61 ± 0.39	3.00 ± 0.32	3.20 ± 1.94	3.34 ± 0.53	3.98 ± 0.92
Total bilirubin (U/L)	<2.00	<2.00	<2.00	<2.00	<2.00	<2.00
Alkaline phosphatase(mmol/l)	143.42 ± 12.74	134.23 ± 3.08	144.73 ± 18.08	131.12 ± 4.50	145.0 ± 20.40	126.9 ± 5.29
Urea (mmol/l)	7.20 ± 0.15	7.19 ± 0.29	6.30 ± 0.2	6.33± 0.22	6.00 ± 0.23	5.19 ± 0.90*
Potassium (mmol/l)	7.50± 0.07	5.92 ± 0.11	6.10 ± 0.04	5.55 ± 0.09	7.50± 0.05	6.36 ± 0.68
Sodium (mmol/l)	149.00 ± 0.12	135.53 ± 0.92	147.50 ± 0.40	139.33 ± 1.10	147.80 ± 0.41	144.33 ± 1.00
Chloride (mmol/l)	116.14 ± 3.29	100.33 ± 0.13	109.00 ± 2.93	100.00 ± 0.50	110.00± 2.10	111.39 ± 1.09
Creatinine (μmol/l)	26.00 ± 1.14	27.11 ± 1.11	27.66± 1.00	27.77 ± 1.10	29.90 ± 1.97	27.00 ± 1.23
Uric acid (μmol/l)	0.17 ± 0.02	0.19.5± 0.06	0.18 ± 0.05	0.20 ±0.05	0.27 ± 0.09*	0.20± 0.09
Total cholesterol(mg/dl)	169.30±9.80	131.33±7.22	122.58±11.10	119.12±5.05	133.00±19.99	99.20±9.90*
Triglyceride (mg/dl)	155.32±6.44	144.50±13.7	132.40 ±7.68	153.30±11.4	129.90±10.50	123.80±7.25
HDL cholesterol (mg/dl)	76.22±6.04	66.80±10.50	68.90±11.30	65.60±11.61	75.52±12.58	71.15± 13.55

Values are presented as mean ± SD, (*P < 0.05)

### Morphological parameters

Microscopic examination of liver, spleen, kidney, lung, pancreas, brain, and heart cross sections did not exhibit any obvious changes in the color or the texture when compared to those of the control group. The organ weight was calculated per animal at the time of euthanasia. The related data are depicted in Table 3. The microscopic examination of organs indicated the presence of fat in the liver of females in both treated groups with EOs, whereas the liver of male mice treated with EO did not show any alteration (Fig. 2.2). Furthermore, the kidneys of male mice treated with EO did not show any changes, but slight lymphocytic infiltrate was observed in the kidneys of female mice treated with EOs (500 mg/kg) (Fig. 2.3). Other than that, lung (Fig. 2.4) and spleen (Fig. 2.5) histology of the treated mice exhibited a normal architecture structure as found in the control group (Figs. 3, 4 and 5).

**Fig. 2:**
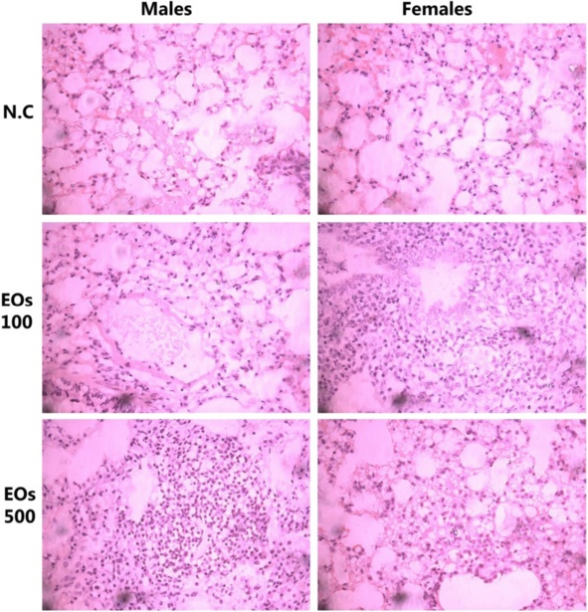
Representative microscopic findings for the liver section of Swiss mice, males (left) and females (right) treated orally with 100 and 500 mg/kg essential oils EOs or negative control N.C (water + 1 % Tween-80) for 28 days. The methods applied hematoxylin and eosin method (400 x)

**Fig. 3:**
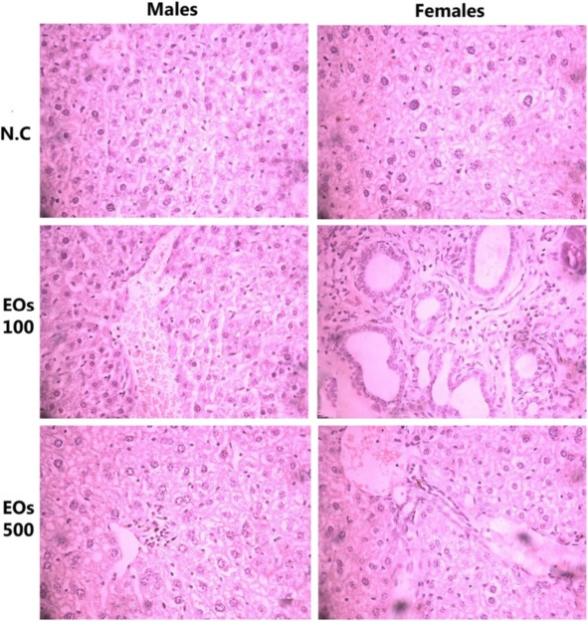
Representative microscopic findings for the kidney section of Swiss mice, males (left) and females (right) treated orally with 100 and 500 mg/kg essential oils EOs or negative control N.C (water + 1 % Tween-80) for 28 days. The methods applied hematoxylin and eosin method (400 x)

**Table 3:** Effects of the subchronic oral administration of EOs on organ weights in Swiss mice

Organ weight (g)	Control		EOs 100 mg/kg		EOs 500 mg/kg	
	Male	Female	Male	Female	Male	Female
Brain	0.40 ± 0.03	0.45 ± 0.07	0.40 ± 0.02	0.46 ± 0.02	0.40 ± 0.03	0.45 ± 0.03
Heart	0.25 ± 0.02	0.19 ± 0.01	0.24 ± 0.03	0.18 ± 0.01	0.24 ± 0.04	0.19 ± 0.01
Liver	2.36 ± 0.55	2.28 ± 0.55	2.22 ± 0.10	2.56 ± 0.19	2.34 ± 0.08	3.10 ± 0.21*
Spleen	0.25 ± 0.02	0.20 ± 0.02	0.20± 0.02	0.21± 0.02	0.20 ± 0.02	0.23 ± 0.02
Kidney	0.29 ± 0.01	0.32 ± 0.01	0.26 ± 0.1	0.25 ± 0.1	0.24 ± 0.03*	0.23 ± 0.07*
Lungs	0.25 ± 0.02	0.23± 0.02	0.24 ± 0.02	0.22 ± 0.02	0.23 ± 0.02	0.24 ± 0.03
Pancreas	0.30±0.05	0.35±0.04	0.29±0.03	0.36±0.02	0.31±0.04	0.36±0.04

Values are presented as mean ± SD, (*P < 0.05)

**Fig. 4:**
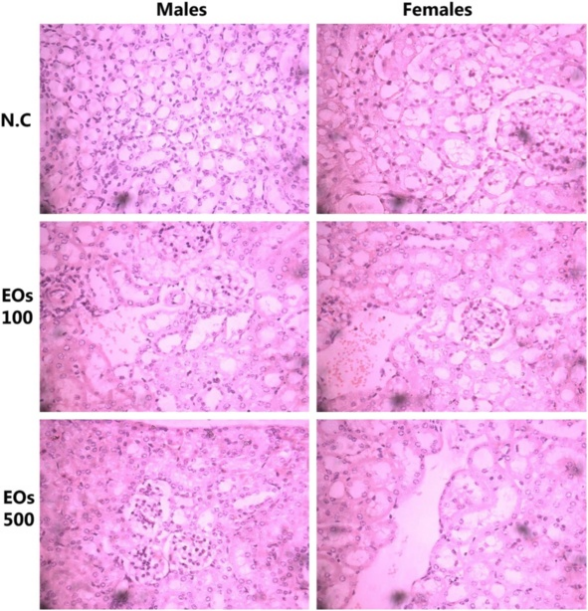
Representative microscopic findings for the lung section of Swiss mice, males (left) and females (right) treated orally with 100 and 500 mg/kg essential oils EOs or negative control N.C (water + 1 % Tween-80) for 28 days. The methods applied hematoxylin and eosin method (400 x)

**Fig. 5:**
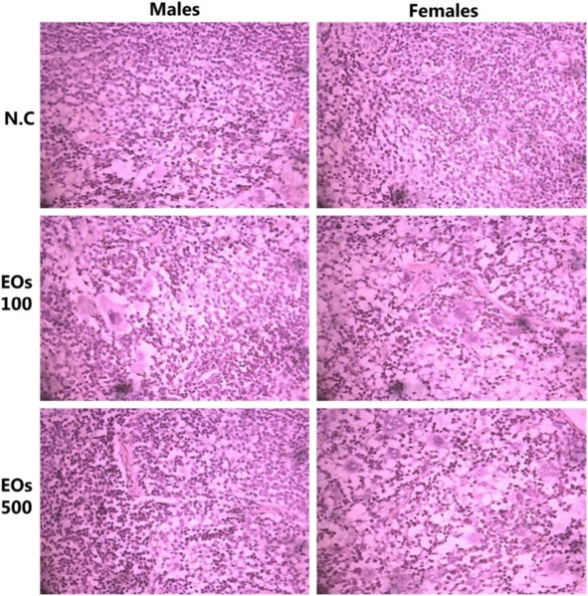
Representative microscopic findings for the spleen section of Swiss mice, males (left) and females (right) treated orally with 100 and 500 mg/kg essential oils EOs or negative control N.C (water + 1 % Tween-80) for 28 days. The methods applied hematoxylin and eosin method (400 x)

### In vivo anti-tumor study of A. crassna essential oil towards colorectal cancer

**Fig. 6:**
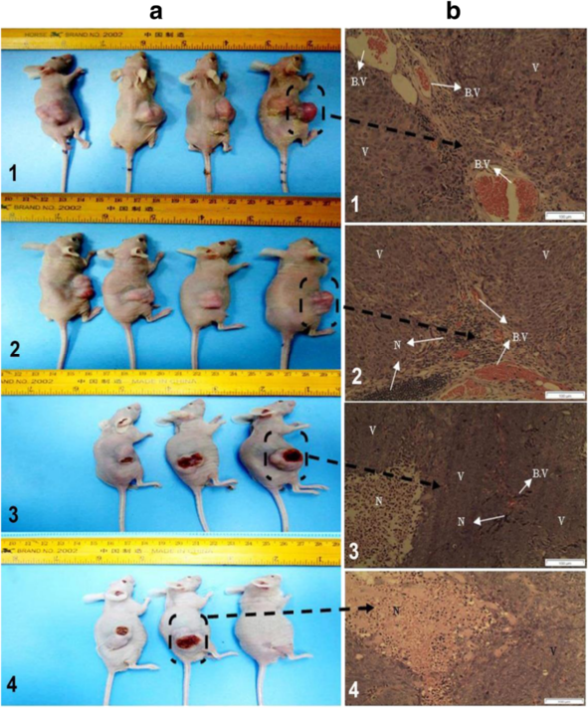
The subcutaneous tumors in NCR nude mice (**a**): Untreated group (1), treated group with EOs at 50 mg/kg (2), 100 mg/kg (3), 200 mg/kg (4). Cross sections of tumor tissues (**b**): untreated animals (1), treated animals with 50 mg/kg (2), 100 mg/kg (3), 200 mg/kg. The tissues were stained with Hematoxylin-eosin. (B.V) refers to blood vesicles, (N) refers to necrotic cells and (V) refers to viable tumor cells

**Fig. 7:**
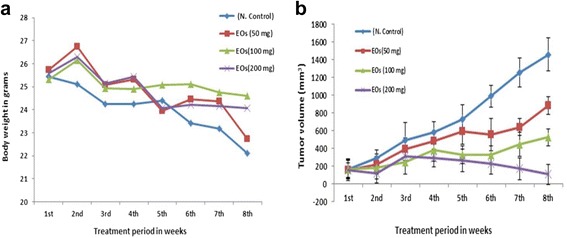
**a** Body weight changes of nude mice during the 8 weeks of anti-tumor study. **b** Analysis of tumor size versus time (days) after treatment with the EOs extracts compared to the untreated group. Values are presented as mean ± SD

The in vivo anti-colon cancer effect of the EOs extract was investigated on the HCT 116 subcutaneous tumor model established in NCR nu/nu nude mice. The results are presented as average tumor size ± SD (n = 6). The treatment with the EOs extract caused significant reduction in the tumor size compared to those in the untreated group (Fig. 6-a). Moreover, data analysis was performed by considering the tumor size recorded in the first week interval and showed that significant reduction in tumor size was achieved after 8 weeks. Nonetheless, a significant ∆T/∆C = 0.37 %( P < 0.05) anti-tumor activity of EOs (100 mg/kg) on 8-week post-cell inoculation day had been recorded. At a dose of (200 mg/kg) on the sixth week post–cell inoculation day, the EOs showed profound activity (0.24 %, P < 0.01). Moreover, the analysis of the tumor cross sections revealed apparent differences in the extent of necrotic regions between the treated and untreated tumors (Fig. 6-b). In addition, the histological feature of the tumors excised from untreated animals showed a high number of blood vessels with less necrosis and apoptosis with a compact layer of cells. Meanwhile, in animals treated with EOs, the tumor tissues showed loss of cell compactness and severe necrosis with areas of low density of blood vessels, as well as many pools of tumor cells. The changes in body weight and tumor volume are depicted in Fig. 7.

## Discussion

One of the major obstacles in the use of medicinal plants is the lack of scientific evidence and clinical data about their efficiency and safety assessment. However, traditional use does not ensure the safety and effectiveness of these mixtures. This is the reason for this study to look into the acute and sub-acute toxicity of the hydrodistillation extract of *A.crassna* EOs on Swiss mice. The results obtained from the acute test did not induce toxicity signs in the mice after single administration of EOs at the dose of 2000 mg/kg. This suggests that LD_50_ more than 2000 mg/kg exhibited a characteristic of low toxicity [22]. Consequently, sub-chronic toxicology study was conducted in order to determine the long-term effect of EOs in animals. Oral administration at repeated-doses (28 days) of the EOs in mice of both sexes did not reveal any signs of morbidity or mortality. Moreover, there was infrequent change in food and water consumptions among the treated groups of both sexes, but these changes did not affect the weights of the body and the organs of the mice during the treatment period (Fig. 1). Furthermore, it showed that some extracts from plants have the ability to alter body weight gain and organ weight parameters through their cumulative effects. Any changes of these parameters indicate a toxic effect of the plant extracts [23]. With that, the results suggested that the EOs extract of *A.crassna* did not produce any side effect on these parameters. During the sub-chronic toxicity investigation, a significant ($P$ < 0.05) increase in total red blood cells (RBC) in female animal treated with 500 mg/kg was observed in hematological analysis; suggesting that the oral administration of EOs had no effect on the circulating peripheral blood cells or on their production. However, the total white blood cells (WBC) displayed a slight decrease in female animal treated with 500 mg/kg; indicating the role of daily administration of EOs in weakening the immunological activity of Swiss mice [24]. Meanwhile, in the analysis of biochemical parameters, the daily oral administration of the EOs extract did not exhibit any changes in total protein, albumin, creatinine, urea, total cholesterol, and triglyceride in the treated groups of both sexes. Only male animal treated with EO of 500 mg/kg had a significant increase ($P$ < 0.05) in the level of uric acid when compared to the control group. In the macroscopic appearance of the organs, animals treated with EOs did not produce abnormal signs in internal organs. Only the female mice, nonetheless, showed an increase in the liver relative weight and a decrease in the kidney relative weight. Moreover, fat was detected in the liver and discrete lymphocytic infiltrate in the kidneys of female mice treated with EOs. It is possible that the increase in liver relative weight among female mice had been due to the presence of fat. However, no side effect was found on the usual biomarkers of liver and kidney toxicity; suggesting that EOs did not cause adverse effect to these organs. Generally, the boundary between toxic and non-toxic extracts depends on several aspects, such as the strength of secondary metabolites, the quantity consumed, part of the plant, and the extraction method.

Commonly, the aqueous extracts seem overall to be less toxic than organic extracts, as the relative low toxicity in the aqueous extracts relies on the fact that they contain a wide class of phytochemical components in a similar way to their existence in natural form and hence, exhibit less risk of side effect [23]. The chemical analysis of hydrodistillation extract for *A.crassna* stem bark also demonstrated versatile classes of sesquiterpenes and polyphenols [15], and this is also true for the species of Aquilaria genus, in which these phytochemical components were identified as the main constituents [25]. On top of that, it has been reported that plant-derived extracts containing a wide range of phytochemicals constituents that exhibit strong cytotoxicity against cancer cells, and the major part of this activity is from the additive and the synergistic effects of phytochemicals in the plant extract [26, 27]. One obvious drawback in medicinal plants research, however, is that the majority of its biological activity is conducted *in vitro*, with little regard for the *in vivo* study. Thus, it is essential to have credible experimental models that can mimic the native tumor microenvironment. This is the key point when aiming to translate data from *in vitro* to *in vivo* and from traditional point of view to pre-clinical application [28]. In parallel with our previous *in vitro* data, the second significant findings has significantly been associated to the *in vivo* anti-tumor effects of EOs using an established colorectal cancer model of subcutaneous xenograft. As a result, we found that the growth of colorectal tumors in nude mice was significantly inhibited by EOs treatment for 8 weeks, with 200 mg/kg daily dosage being the most effective dosage. Indeed, several lines of evidence indicated that plant-derived bioactive compounds can be exploited to regulate the cancer cells by targeting multiple signaling pathways that are implicated in the consolidation and the maintenance of tumor microenvironmental features [29, 30]. Besides, the anti-tumor activity of EOs may be explained due to the cytotoxicity effect on the colorectal cancer cell HCT 116 as evident by the morphological feature of shrinking tumor, as well as the presence of necrotic/apoptotic regions in subcutaneous tumors of treated animals, or due to the reduction found in the intratumor blood vessels, as evident by the remarkable reduction in tumor vascularization in treated groups.

## Conclusion

The present study demonstrated that EOs is safe with LD_50_ above 2000 mg/kg. The histopathological variations revealed no clinically relevant changes, since they occurred in non-generalized patterns in both treated mice and control. Moreover, the results highlighted the possible application of EOs in colorectal cancer prevention/treatment and further laid a solid foundation for pre-clinical use in animal.
